# Tubular lipid binding proteins (TULIPs) growing everywhere^☆^

**DOI:** 10.1016/j.bbamcr.2017.05.019

**Published:** 2017-09

**Authors:** Louise H. Wong, Tim P. Levine

**Affiliations:** UCL Institute of Ophthalmology, 11-43 Bath Street, London EC1V 9EL, UK

**Keywords:** BPI, bacterial permeability-inducing protein, CETP, cholesterol ester transfer protein, CFTR, cystic fibrosis transmembrane conductance regulator, DALI, distance matrix alignment, DUF, domain of unknown function, ENaC, epithelial sodium channel, ER, endoplasmic reticulum, ERMES, ER-mitochondrial encounter structure, E-Syt, extended-synaptotagmin, JH, juvenile hormone, JHBP, JH binding protein, LTP, lipid transfer protein, LPS, lipopolysaccharide, LBP, LPS-binding protein, LAM, LTP anchored at a membrane contact site, PC, phosphatidylcholine, PS, phosphatidylserine, PLTP, phospholipid transfer protein, PLUNC, palate lung and nasal epithelial clone, RMSD, root mean square distance, SMP, synaptotagmin mitochondria and lipid binding protein, TULIP, tubular lipid binding protein, Anacetrapib, Cystic fibrosis transmembrane conductance regulator (CFTR), Endoplasmic reticulum-mitochondria encounter structure (ERMES), Extended-synaptotagmin (E-Syt), Lipid traffic, Lipopolysaccharide (LPS), PDZK8, Tether

## Abstract

Tubular lipid binding proteins (TULIPs) have become a focus of interest in the cell biology of lipid signalling, lipid traffic and membrane contact sites. Each tubular domain has an internal pocket with a hydrophobic lining that can bind a hydrophobic molecule such as a lipid. This allows TULIP proteins to carry lipids through the aqueous phase. TULIP domains were first found in a large family of extracellular proteins related to the bacterial permeability-inducing protein (BPI) and cholesterol ester transfer protein (CETP). Since then, the same fold and lipid transfer capacity have been found in SMP domains (so-called for their occurrence in synaptotagmin, mitochondrial and lipid binding proteins), which localise to intracellular membrane contact sites. Here the methods for identifying known TULIPs are described, and used to find previously unreported TULIPs, one in the silk polymer and another in prokaryotes illustrated by the *E. coli* protein YceB. The bacterial TULIP alters views on the likely evolution of the domain, suggesting its presence in the last universal common ancestor. The major function of TULIPs is to handle lipids, but we still do not know how they work in detail, or how many more remain to be discovered. This article is part of a Special Issue entitled: Membrane Contact Sites edited by Christian Ungermann and Benoit Kornmann.

## Highlights

• Proteins with the tubular lipid binding fold exist in a wider variety than is usually appreciated.
• TULIPs are found in prokaryotes, altering views on their evolution.
• It is not yet known whether TULIPs transfer lipids as tunnels or as shuttles.
• Tests have not yet been done to say if TULIPs with SMP domains (for example E-syts and ERMES components) tether contact sites.
• It is likely that more TULIPs remain to be discovered.

## 1 Introduction

Evolution has created many situations in which hydrophobic, water-insoluble molecules such as lipids are transported through the aqueous phase by proteins. Lipid transport outside cells is important for scavenging or detecting lipids within the environment. Lipid transport inside cells is required to move lipids between membrane-bound compartments, even if these compartments are linked by vesicular traffic [1]. The lipid transfer proteins (LTPs) that mediate lipid transport both inside and outside cells have often been discovered through purification of an activity that carries lipid between artificial bilayers *in vitro* [2]. Structural studies of these LTPs show that they all possess a cavity that engulfs the hydrophobic portion of a lipid, if not the entire lipid molecule [3]. The number of lipids that organisms deal with is large, and the non-vesicular routes inside cells are also numerous, so it is interesting to find that over 100 LTPs are encoded in a mammalian genome [4], and that 17 different protein folds can act as LTPs for lipids [3]. This review describes the Tubular lipid binding proteins (TULIPs), a large superfamily of LTPs with a distinctive tubular fold [5]. Note that “family” is a technical term referring to a group of protein homologues that can be linked together by applying conventional sequence alignment tools, such as PSI-BLAST or PFAM. In contrast for TULIPs as a whole the correct term is “superfamily”, which indicates multiple families sharing sequence similarity that is not detected by conventional tools, but is detected by more sensitive sequence comparison methods. Thus, new TULIP domains have been found that had could not have been identified by sequence homology with known domains. The discovery of TULIP structures in bacteria that is reported here shows that the superfamily is even more widespread than previously thought.

## 2 Classifying the full range of TULIPs

### 2.1 The main varieties of TULIP

Some of the first LTPs ever described are now known as founding members of the TULIP family [6], [7]. These are extracellular mammalian proteins that solubilise a range of hydrophobic molecules, including vitamins, phospholipids and neutral lipids such as cholesterol ester and triacylglycerols. Both cholesteryl ester transfer protein (CETP) and phospholipid transfer protein (PLTP) transfer lipids between lipoproteins in the extracellular fluid. Two other members of this family bind lipopolysaccharide (LPS), the major lipid of the outer membrane of Gram-negative bacteria that induces toxic shock. One is the plasma protein LPS-binding protein (LBP), which mediates innate immune responses to LPS by presenting it to monocytes [8], [9]. The other is Bacterial Permeability-Increasing protein (BPI) found in azurophilic granules of neutrophil polymorphs, which has a complex oxygen-independent bactericidal action that arises from LPS binding [10], [11].

**Fig. 1:**
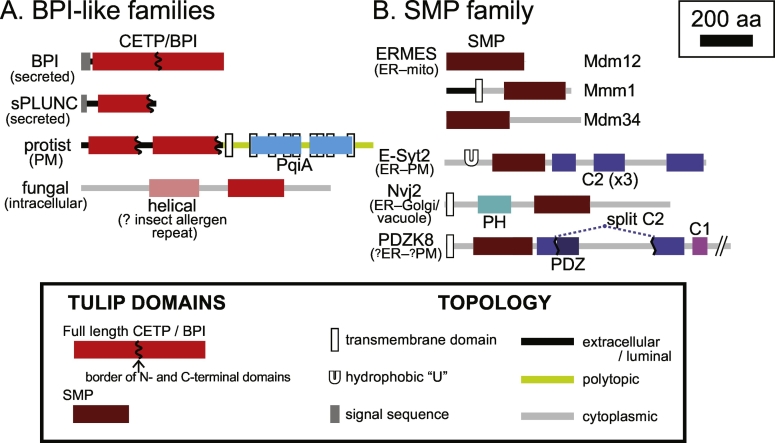
Domain structure of TULIP proteins. TULIP domains are found in the two major families: CETP/BPI domains (A) and SMP domains (B). Family and membrane topologies (cytoplasmic *vs.* polytopic *vs.* secreted to become extracellular or luminal) are indicated as shown in the key. Location and accompanying domains are marked in the figure. The SMP proteins shown here are representatives of the four types that are widely spread through eukaryotes [20]. The fungal protein shown is SPAC32A11.02c from *S. pombe*. Its entire N-terminus is predicted to be helical, with residues 232–401 aligning well in HHsearch with residues 44–172 of Bla g 1, a helical insect allergen repeat protein crystalised as 4jrb. HHsearch does not make this prediction definitively, and other all-helical hits include bimodular sensor domains, which typically bind chemo-attractants. The C2 domain in PDZK8, split so that its two segments can assemble into one complete domain, was missed previously [34].

CETP, PLTP, LBP and BPI (hereafter referred to as CETP/BPI) were originally identified for their lipid solubilizing and transfer activities, with their TULIP folds discovered subsequently by X-ray crystallography. CETP/BPI are founders of a much wider protein family of sequence homologues that include the palate lung and nasal epithelial clone (PLUNC, also called BPI fold-containing family, BPIF) proteins. These are highly abundant components of the secretions of upper airways, nasopharynx and tears of mammals [12], [13], [14] and of egg albumen in birds [15], [16]. In addition, there are full length homologues (homology spanning > 450 aa) widely dispersed in all branches of eukaryotic evolution, from fungi to protists, algae and plants. For example, CETP/BPI homologues are found in ascomycetes, even though they are missing from *S. cerevisiae*. All CETP/BPI family proteins are extracellular, and most are secreted proteins 350–500 aa long (Fig. 1A). However, a small number of protist and amoebal proteins are anchored in the plasma membrane by a C-terminal PqiA domain. This domain, which is usually found in prokaryotic proteins, has 9–12 transmembrane domains and a proposed function as a lipid permease (Fig. 1A) [17], which suggests that the associated CETP/BPI domains may handle the same lipid ligands as the PqiA domain.

### 2.2 Stumbling across double TULIPs and identifying more singles

In addition to the proteins related to CETP/BPI by BLAST and PSI-BLAST, there are some whose similarity was only discovered when their structures were solved. For any newly solved structure, the Cα backbone can be aligned in three dimensions to all other structures by tools such as DALI (distance matrix alignment) [18]. The first and most important discovery was made by solving the protein structure of one member of the family. This revealed that every CETP/BPI family member contains a large internal repeat [19], meaning that the basic unit of “TULIP-ness” is not the whole of CETP/BPI, but just half of it. Each half, *i.e.* both the TULIP-N and the TULIP-C domains on their own, have LTP activity and they each have tubular structures with separate lipid binding cavities (see next section). Thus, most CETP/BPI family members contain two LTPs as a tandem hetero-dimer. The main variation on this pattern is in the PLUNCs, where the TULIP-C domain can be missing, leaving just one domain (short PLUNC) (Fig. 1A) [20].

Another part of the CETP/BPI family that was originally identified purely from solved structures is found in arthropods, which appeared to have no sequence homologues of CETP/BPI. The first arthropod TULIPs identified were the juvenile hormone (JH) binding protein (JHBP) in haemolymph [21], [22], and the regulatory protein Takeout [23], the structures of which turned out to be super-imposable on TULIP-N domains from CETP or BPI. JHBP and Takeout have hydrophobic ligands (JH and isoprenoids respectively), which the proteins bind in the extracellular milieu and deliver to cells, similar to the way LBP presents LPS. The same arthropod family includes the group 7 allergens, for example the house dust mite *Dermatophagoides pteronyssinus* allergen Der p 7. These are secreted proteins that may function in binding and presentation of polymyxin B, a bacterial lipopeptide, to the innate immune system [24]. As for the link between CETP/BPI and SPAC32A11.02c, Der p 7 is clearly in the same family as JHBP and Takeout, although PSI-BLAST searches must be initiated with Der p7, not JHBP, to reveal this (data not shown).

### 2.3 Profile-profile searches find TULIPs inside cells

The discovery of the two structurally similar domains within CETP/BPI indicated that an ancient duplication of the domain took place, followed by such wide sequence divergence as to make the homology almost invisible, although convergent evolution cannot be formally excluded [20], [25]. Homologies within the TULIP superfamily can be completely missed by conventional sequence alignments. However, there are more sensitive tools that compare profiles of the homologues of a seeded sequence with pre-prepared libraries of similar profiles of possible target sequences. Such profile-profile tools can be many orders of magnitude more sensitive than PSI-BLAST [26], being particularly sensitive where a domain contains an α/β mix [27]. One powerful profile-profile tool is HHsearch, which relies almost entirely on sequence (89%), but also aligns solved or predicted secondary structure (weighted 11%) [28]. This has been used to successfully predict distant homologues of CETP/BPI [29].

The proteins that profile-profile searches find first, *i.e.* those closest to the core CETP/BPI family, are in fungi (for example the SPAC32A11.02c protein in *S. pombe*, Fig. 1A) and phylogenetically diverse organisms from protists to amoebae [5], [20]. They are 500–1200 aa long with a single TULIP domain near the C-terminus, and most significantly they are intracellular. Although PSI-BLAST seeded with CETP/BPI finds none of these, if PSI-BLAST is seeded with SPAC32A11.02c, hits are obtained to BPI-N domains from the second iteration onwards (data not shown), so they are all in the same protein family. The region preceding the CETP/BPI domain has not been described previously, but it is predicted by HHsearch to be mainly α-helical, with a suggestion that it is homologous to the “insect allergen repeat”, an LTP previously thought to be confined to insects (Fig. 1A) [30].

The largest group of homologues to CETP/BPI identified by HHsearch are the synaptotagmin mitochondria and lipid binding protein (SMP) domains [29]. The prediction that SMP domains are homologous to TULIPs such as CETP/BPI has since been verified by crystal structures of three SMP domains [31], [32], [33]. SMP domains had been identified throughout eukaryotic evolution in moderately large numbers per genome (human 7, yeast 7, plant 11), with various accessory domains also present [34]. The key accessory domains are transmembrane domains, which are almost ubiquitous, meaning that SMP proteins are membrane-anchored (Fig. 1B). The exceptions are Mdm34 and Mdm12, which are part of the endoplasmic reticulum (ER)-mitochondrial encounter structure (ERMES) complex. ERMES also contains Mmm1, an SMP protein anchored in the ER, Mdm10, a porin embedded in the mitochondrial outer membrane and the small Miro-like GTPase Gem1 [35], [36]. Thus, all SMP domains are in proteins anchored to the cytosolic face of a membrane. More specifically, all SMP proteins target membrane contact sites where two organelles come very close to each other (see Box 1) [37], which is likely to be functionally important.

Box 1 **Membrane contact sites.**

The architecture of the intracellular world has gone through a quiet revolution in the last decade, with an increased recognition that most organelles interact physically with each other at contact sites [56], [76], [104]. This also applies to the pair of membranes in Gram-negative bacteria, as well their descendants in mitochondria and in chloroplasts. These physical proximities allow metabolic channelling of material and information between any pair of compartments. The material that is passed between organelles at contact sites varies from large molecules, such as motor proteins, to small molecules, such as calcium ions and lipids. The evidence that supports a role for contact sites in lipid traffic is mainly circumstantial: many LTPs are enriched at contact sites [76], [105]. Even though a full demonstration of LTPs shuttling lipids across a contact site is still lacking, it is an appealing model that the targeting of LTPs to different contact sites determines their ability to mediate intracellular lipid traffic. It has also been calculated that confining lipid traffic to contact sites increases efficiency because the diffusional step, which may (or may not) be rate-limiting, is 1000 × faster in small cells and even more in larger cells [106]. Targeting of some LTPs to multiple contact sites is a variation that may allow them to function on multiple routes [107], [108], [109].

Alt-text: Box 1

### 2.4 TULIPs in prokaryotes

TULIPs were originally identified in the extracellular spaces of eukaryotes, and subsequently shown to also be widespread inside eukaryotic cells. But the literature indicates they are absent from prokaryotes [20]. A deeper mining of all data resources shows that they are present, not absent. A prokaryotic TULIP structure was deposited in the Protein Data Bank in 2010 without a reporting publication: the *E. coli* protein YceB (PDB accession code 3L6I) [38]. By DALI, YceB aligns with the TULIP-C domain of CETP/BPI with a Z-score = 9.2 (153 residues align with root mean square distance (RMSD) 4.0 Å). For comparison, this alignment is stronger than that between TULIP-N and TULIP-C (Z-score is 6.0 across 159 residues, RMSD = 2.7 Å; note that higher Z values indicate greater similarity) [39]. The YceB structure has been mentioned in passing for its similarity to short-PLUNC1 [40], but it has not been reported in detail. The Cα backbone of YceB is virtually superimposable on TULIP-C of BPI, even though the degree of identity is only 5% (Supplemental Movie 1). The degree of sequence similarity is low, but the majority of YceB makes weak matches in HHsearch to multiple eukaryotic TULIPs (data not shown), which is consistent with divergent evolution, although convergent evolution is not excluded. This indicates that there is an additional family of TULIPs found in prokaryotes and that the TULIP fold is older than has generally been appreciated (Table 1).

**Table 1:** Evolutionary origins of 17 LTP (super)-families.

7 found in prokaryotes:
lipocalin*, LppX‡, LptACD‡, NTF-2*, SCP-2°, StAR-kin°, TULIP°¶
10 not found in prokaryotes:
CRAL/TRIO (Sec14), elicitin, FAD/NAD binding, GLTP, insect allergen repeat, NPC1N, NPC2/MD2, ORP, plant nsLTP, saposin

LTPs that can transfer lipids fall into 17 superfamilies [3], seven of which are found in prokaryotes: ‡LTPs in prokaryotes only; °LTPs also in eukaryotes; *LTPs also in eukaryotes but the ligands for all the eukaryotic members are smaller than lipids; ¶prokaryotic TULIPs have not been described in detail previously.

## 3 Structure-function relationships among the TULIPs

The distinctive tubular structure of TULIPs has been considered to have significant physical implications. Here different aspects of the structure-function relationship are addressed.

### 3.1 Always tubular on the outside but not always tubular on the inside

**Fig. 2:**
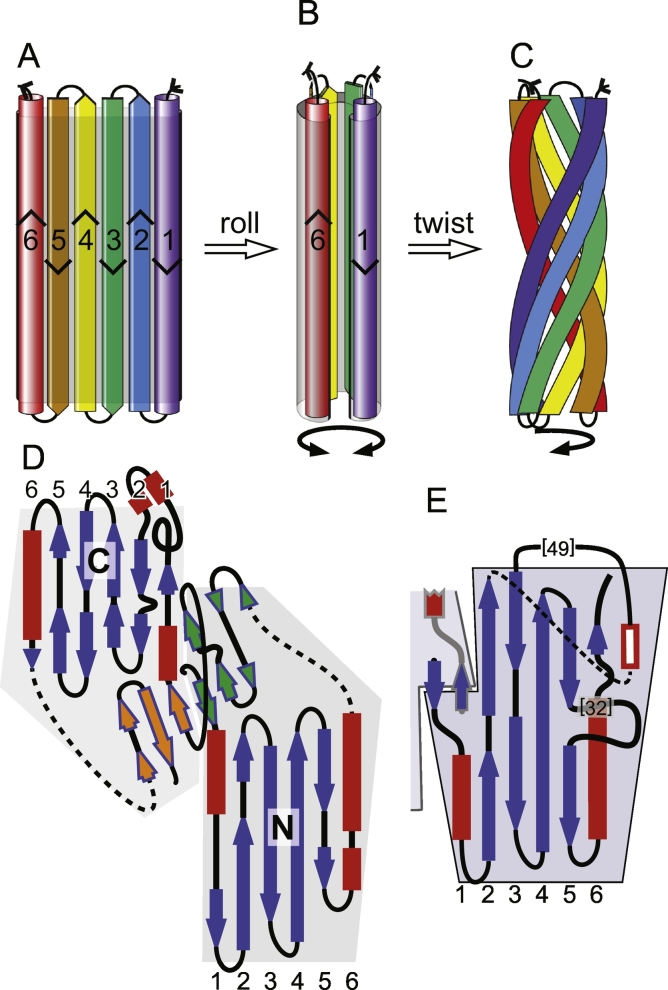
How TULIPs are made. A–C: TULIPs contain 6 elements (coloured in a pseudo-rainbow blue to red), which form an anti-parallel meander (A) that is rolled up (B) and twisted (C) to form a super-roll. The head of the molecule, where both ends of the polypeptide are found, is shown at the top. D and E: structural topology maps of BPI N- and C-terminal domains (D) and one Mdm12 domain (E), including the dimerisation interface of a second domain (highlighted in grey). α and β elements (numbered as in A) are coloured red and blue respectively. Dotted lines indicate loops that have been extended for the purpose of aligning elements in 2D. Inserted loops in Mdm12 are shortened with their total number of residues shown in brackets and any structural element they contain filled in white. Background shapes show the extent of each domain and the dimerisation interface. For BPI, this interface consists of a 6 ½ strand β-meander contributed almost equally by both domains (elements filled green and orange). For Mdm12 dimerisation is mediated by a short N-terminal β-sheet before the first element [32]. The same is presumed to apply to Mdm34, although it has not yet been crystalised, as it shares a highly similar N-terminus. For E-Syt2, the contact interface is formed by conserved residues in the loops, which may allow heterodimerization (not shown) [31].

The N- and C-terminal TULIP domains of CETP/BPI are described as tubular in part because they are cylindrical, being 2–3 times longer than they are wide (dimensions ~ 7 × 2.5 × 2.5 nm, Fig. 2A–C). The core of each domain consists of ≥ 175 residues that make up 6 antiparallel structural elements (Fig. 2A). Structural elements 2–5 are a β-meander. Elements 1 and 6 always contain some α helix, but can be mixed α/β, especially the 1st element. The αββββα elements form a fold described as a super-roll. The way this is produced can be visualised in steps: the 6 elements are arranged in an anti-parallel sheet (Fig. 2A). The sheet is rolled into an incompletely closed tube, with hydrophobic residues in the middle (Fig. 2B). If the internal side chains are small enough then there will be cavities for lipids to bind. Finally, the tube is twisted into a tubular spiral (Fig. 2C). Superhelical twisting of long α-helices in the 1st or 6th elements requires breaks in their helical structure. Similarly, twisting of the β-sheets requires breaks from the parameters that stipulate β-sheet, creating multiple β-bulges [19], [22]. Finally, many but not all TULIPs form head-to-head dimers. For SMP domains this is achieved not only by head-to-head heterodimerization between two different SMP molecules [32], but also by homodimerisation [31], [32], [33]. CETP and BPI dimers are crescents 13 nm long.

Given this general form, there are still significant variations between TULIP domains. Firstly, there is the difference between TULIP-N and TULIP-C. At the sequence level they share no significant homology. However, residues at analogous positions in the two domains share biochemical environments, as defined by a combination of secondary structure, solvent accessibility and local polarity [25]. The secondary structural topology maps of TULIP-N and TULIP-C domains are very similar (Fig. 2D). The dimerisation interface (the “head” of each domain) in CETP/BPI is quite elaborate, consisting of multiple β-sheets from each side. The dimerisation interfaces are much simpler for SMP domains, which also differ from other TULIPs in the secondary structures of the 1st and 6th elements (Fig. 2E).

**Fig. 3:**
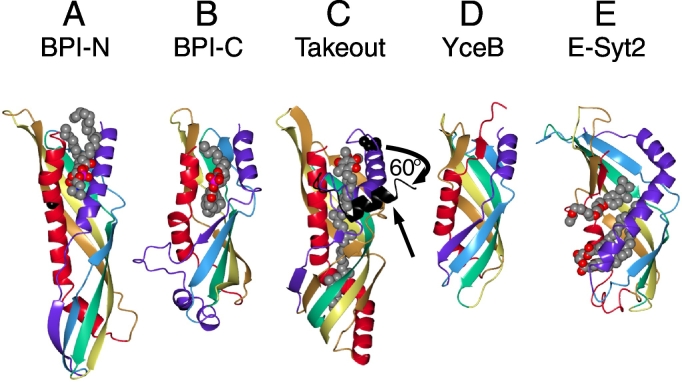
Solved TULIP Structures. Five different TULIP domain structures A: BPI N-terminus, B: BPI C-terminus, C: Takeout, D: YceB, E: E-Syt2, with the heads of the molecules, where both ends of the polypeptide are found, at the top as in Fig. 2. The six structural elements are coloured in a pseudo-rainbow as in Fig. 2A-C. Ligands are coloured with carbon in grey and other atoms as per convention. In C, the arrow indicates the alternate position of helix 1 (drawn in black) that rotates 60 °C in the apo-form of JHBP, a Takeout homologue. In all 5 cases, similarity of fold was demonstrated by structural techniques, not by PSI-BLAST. Despite similarities, there are minor differences: BPI-N has the 5th and 6th elements attached by a disulphide bond; BPI-C has a helix-turn-helix insert after the 1st element; Takeout has a loop inserted in the 1st element, YceB is smaller than the others and the helix in its 6th element is followed by a short sheet; the latter feature is also found in E-Syt2, where the 1st element is entirely helical. PDB files (A/B = 1 bp1; C = 3e8t and residues 18–41 of 3aot; D = 3l6i and E = 4p42) were visualised with CCP4MG software (version 2.10.4).

Considering just the core domains, there are several small structural differences between TULIP-N and TULIP-C of BPI (Fig. 3A and B). An obvious difference is length of the domain, with TULIP-N longer than TULIP-C. Takeout/JHBP are close to TULIP-N in terms of sequence, but for length they are intermediate between TULIP-N and TULIP-C (Fig. 3C), while YceB is shorter than TULIP-C (Fig. 3D). Takeout/JHBP crystalises as a monomer and is presumed to function as a monomer. The cylinders of the SMP domains such as in extended-synaptotagmin 2 (E-Syt2) are slightly wider and also shorter than other TULIPs (Fig. 3E).

While the secondary structural topologies are quite similar, the internal cavity varies considerably. For BPI, both TULIP-N and TULIP-C contain a single phosphatidylcholine (PC) molecule, the position of which differs slightly (Fig. 3A). For CETP, each crystalised domain contains two lipids (PC and cholesteryl ester), and the cavity is larger to allow this. While the PC headgroup projects from CETP, the cholesteryl ester is entirely contained within the cavity, as is found for the ligands of JHBP and Takeout [22], [23]. This is consistent with all the residues lining the internal cavity being hydrophobic. SMP domains bind either one [32], [33] or two phospholipids [31], with headgroups projecting out of the seam between the 1st and 6th elements [31], as seen for CETP/BPI. SMP domains have some specificity for lipid headgroup. For example, Mdm12 is partly selective for phosphatidylcholine or phosphatidylglycerol [32], [41], and Tmem24, a vertebrate-only E-syt-like protein with one C2 domain that is expressed in neurosecretory cells, selects for phosphatidylinositol [33]. However, the extent of selectivity in TULIP domains is less than LTPs that have hydrophilic peptide patches to specifically interact with headgroups [42], [43], [44].

### 3.2 Tunnel models for lipid traffic by TULIPs: minimal movement within an overall static protein

Since TULIPs are mainly beta super-rolls they distantly resemble porins, except there is a complete inversion, since porins are hydrophilic inside and hydrophobic outside, while TULIPs are hydrophilic outside and hydrophobic inside. This has led to suggestions that TULIPs act as relatively static hydrophobic channels (or “tunnels”) through which lipids could flow.

#### 3.2.1 Movements that facilitate lipid exchange

Even if TULIPs do not move *en bloc*, there is the possibility that specific regions move to allow lipid binding. Superficially TULIPs differ from LTPs that fully enclose lipid ligands, such as Sec14. While the latter must undergo rearrangements to allow lipids in and out [45], the lipid molecule is not fully enclosed by TULIPs. So it is conceivable that a TULIP is completely rigid. However, work with insect JHBP indicates that the non-liganded form is flexible and undergoes large shifts on ligand binding. The helix of the 1st element is tightly apposed to the ligand in the holo form. But in the empty (apo) form, the helix bends away from the binding pocket (Fig. 3C) [46]. NMR, which studies proteins in solution, indicates that holo-JHBP is very similar to the crystal structure. But in apo-JHBP 40% of residues, particularly those forming the ligand binding pocket, produced no signals. This indicates high flexibility and multiple conformations around the pocket [46]. Similar movement of a helix near the opening of a TULIP's lipid binding cavity is seen for the C-terminal domain of CETP, where a helix added after the 6th element blocks lipid entry, but has been modelled to enter an open conformation when in contact with HDL [47]. Together these findings indicate that lipid binding by TULIPs requires flexibility and minor rearrangements. Thus, a crystal structure that contains lipid (Fig. 3) is likely to represent just one of many conformations.

#### 3.2.2 SMP domains

A channel–like (tunnel) model has been suggested for phospholipid traffic by SMP dimers [5], with hydrophilic headgroups moving along a continuous seam [29], which is 7 nm long in E-Syt2 (Fig. 2B) [31]. Although other SMPs do not have this long seam [32], [33], the formation of tunnels might still occur in E-Syts. A question here is: do SMP dimers span the gaps where these proteins act? For E-Syt2, ER-plasma membrane contact site gaps have been measured as wider than 15 nm [48], [49], but E-Syt2 dimers are < 10 nm long [31], suggesting that they are unlikely to bridge. This shows the importance of determining if SMP multimers are able to function as one long lipid tunnel that involves dimerisation away from the typical head-to-head interface (see Multimeric assembly below).

#### 3.2.3 CETP

Most work on TULIPs as tunnels has focussed on CETP. Its mode of action is important because CETP inhibitors have been the subjects of expensive (and so far negative) clinical trials to prevent atherosclerosis [50]. An initial model for CETP suggested that cholesteryl esters flow through the tunnel in its crystal structure [51]. However, this occupies < 50% of the length of the pseudo-dimer, making it a poor candidate to act as a tunnel without additional regions of CETP opening up. This has led to many molecular dynamics simulations to predict physiological changes in CETP structure. Simulations have studied the protein alone [52], protein plus lipid contents [53], protein plus HDL [47], [54], and protein plus both donor and acceptor lipoproteins [55]. There is some consensus in the predictions made by these *in silico* studies: the cavities in the N-terminal and C-terminal domains can enlarge (7% in one estimate) [52] and join together across the mid-point of the dimer [53]; loops near the tail of the TULIP can flexibly separate allowing lipid entry to a small cavity that is separate from the main cavity [54], [55]. In the most complex simulation, complete transfer of cholesteryl ester was modelled by applying a direct pulling force on the lipid. Its entry at the tail was followed by further local rearrangements that allowed direct access into the main cavity of the modelled protein [55]. The application of force *in vivo* could be justified here because HDL is predicted to have a higher internal pressure than LDL. These simulations show the attractiveness of the tunnel model. However, simulation is limited because calculations can maximally cover 100–1000 ns, but lipid transfer occurs on a much longer time-scale (10–1000 ms). There are other caveats, described in the section on Lipid transfer.

### 3.3 Shuttle models: protein and lipid moving together

The alternative to the “tunnel model” is that lipids and the TULIP domain surrounding them move together, after forming a LTP-lipid complex. In this “shuttle model” a lipid would be picked up by the TULIP in one place and deposited in another [31], [41]. While some LTPs travel across large cytoplasmic distances, the distance travelled by TULIPs might be only ~ 10–30 nm, which is the gap at most membrane contact sites (see Box 1). SMP domains are mostly anchored by a transmembrane domain, to which they are linked by a quite short unstructured region (Fig. 1B). For E-Syt2 this linker allows the SMP domain to move up to 17 nm from the ER [56]. Thus, all the TULIPs could function by shuttling [57], though whether this model applies *in vivo* has yet to be determined.

### 3.4 Multimeric assembly of TULIPs

**Fig. 4:**
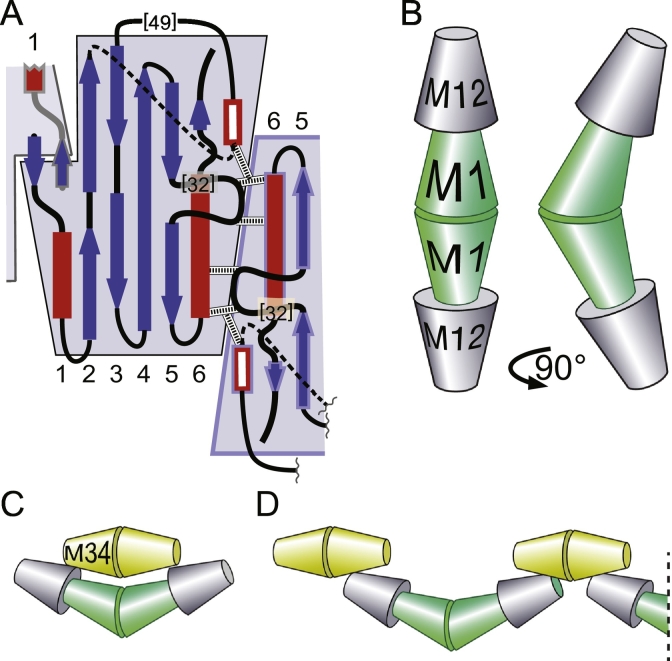
Multimerisation of SMP domains in ERMES. A: Mdm12 can exhibit a second interface for self-interaction. In addition to head-to-head dimerisation (as in Fig. 2E) there is a tail-to-tail self-interaction [32]. Here the helix in element 6 of one monomer forms contacts (white “ladders”) with residues in both the inserted loops of a second monomer (highlighted in light blue); B: Heterotetramers of Mdm12 (grey) and Mmm1 (green) form an extended structure with a central bend of 45°; redrawn from single particle EM visualisations by AhYoung et al. [41]. C and D: Possible hexameric (C) and octameric (D) assemblies of Mdm12, Mmm1 and Mdm34 [41]. Mdm34 (yellow) may homodimerise through interactions of its N-terminal β-sheet, highly similar to that of Mdm12. In addition, it appears to bind Mdm12, which in turn binds Mmm1. Both these interactions are not head-to-head, and they have not yet been studied, though they would allow higher order chains to form.

SMP domains typically homodimerise via the head interface (Fig. 2F). In addition, three SMP proteins in ERMES in budding yeast (*S. cerevisiae*), Mmm1, Mdm12 and Mdm34, form complexes that also include Mdm10 and Gem1. Complexes can contain two copies each of Mdm12 and Mmm1 to make a heterotetramer [32], [41]. Mdm12 also forms homotetramers, which have been crystalised. The crystals contain a TULIP-TULIP interface other than the previously known “head” interface, which involves the helix of the 6th element [32]. There are direct contacts from the helix with a proline-rich loop inserted into the 5th element, and with a short helix in the long loop between elements 2 and 3 (Fig. 4A). These contacts may represent a crystal packing interface, and they have yet to be probed for functional significance. In particular, homotetrameric forms of Mdm12 have not yet been reported by size exclusion chromatography. Even if this interface is physiologically important for Mdm12 self-interaction, it is unlikely to apply to Mmm1, because this lacks the key contacting residues and the inserts found in Mdm12. This means that the molecular basis by which a Mdm12-Mmm1 heterotetramer could form is still unknown [32].

Although the Mdm12-Mmm1 interface is unknown, size-exclusion chromatography shows that Mdm12/Mmm1 heterotetramers are highly elongated [41]. Furthermore, single particle EM together with tagging a maltose binding domain on either the N-terminus of Mmm1 or the C-terminus of Mdm12, showed that the elongated molecule consists of Mdm12-Mmm1-Mmm1-Mdm12, where the heads of Mdm12 bind to the tails of Mmm1 (Fig. 4B) [41]. In addition, an interaction between Mmm1 dimers and Mdm34 dimers requires the presence of Mdm12, which is presumed to lie in between [41]. All of this suggests several possible configurations for the three TULIPs, which have yet to be tested directly (Fig. 4C). The minimal size is a hexamer, but other arrangements might lead to larger complexes, contributing to the concentration of ~ 250 copies of an ERMES subunit in a few puncta in each cell [58]. It is not yet known how any of the non-head-to-head interactions could work in molecular detail. It is possible, but also not yet studied, that similar multimeric assemblies arise between E-Syts at ER-plasma membrane contacts.

## 4 TULIP functions

### 4.1 Lipid scavenging/presentation

One major role of TULIPs is to pre-select hydrophobic ligands for presentation to other proteins, for instance so that the lipids generate signals. A prime example is LBP, which binds LPS in the serum and then activates the immune response by binding CD14 [9]. Presentation is also a property of JHBP, which is secreted into haemolymph, from where it increases the delivery to cells of JH that eventually bind *Met*, inducing nuclear translocation and signalling [59], [60]. LPS is a major target for TULIPs, possibly explaining the presence of BPI homologues throughout eukaryotic evolution, even in single-celled organisms and plants [20]. In humans, as well as being presented to CD14 by LBP, LPS can be inactivated by LBP and PLTP transferring it to serum lipoproteins [61]. The effect of BPI on LPS opposes that of LBP: BPI binds LPS more tightly and prevents presentation to CD14. However, this very tightness of binding allows BPI to disrupt the outer bacterial membrane leading to direct cell damage and increasing the recognition, uptake and killing of bacteria [11]. Given the anti-bacterial properties of this TULIP family, there was interest in developing them as anti-septicaemia therapeutic agents. The entire TULIP-N domain of BPI inhibited Gram-negative septicaemia [62], but the polypeptide is not used clinically [63]. Portions of BPI and other TULIPs have been used as starting points for the design of anti-inflammatory peptides, but the resulting active peptides often only work at high concentration [64], or only bear a distant relationship to the original TULIP [65], [66], so it is not clear if the peptides reflect properties of the whole protein.

### 4.2 Lipid transfer

**Fig. 5:**
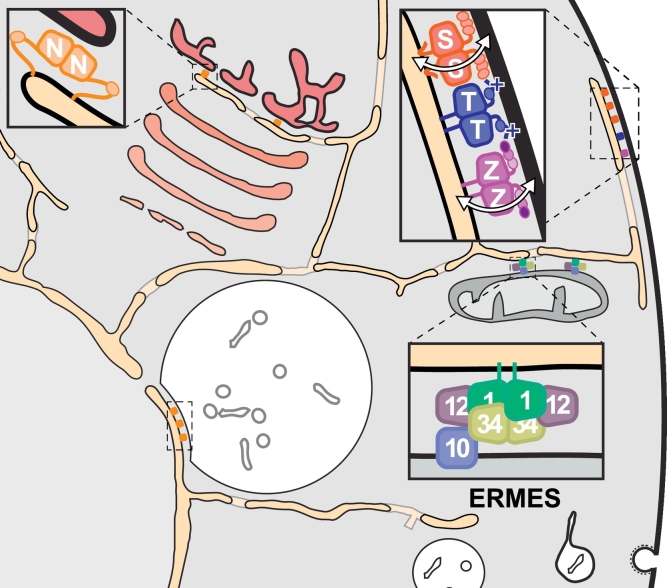
Intracellular targeting of proteins with SMP domains. SMP domain proteins are shown as small coloured rectangles at four intracellular locations, all of which are contact sites that involve the ER. Insets show expanded views at three contacts of SMP and associated domains, all of which are explained in Fig. 1B, except for the polybasic region of Tmem24 (“+”), which is otherwise like E-Syts, though with one C2 domain [33]. The associated domains can bind non-ER membrane determinants including phosphoinositides and other anionic lipids. At ER-mitochondrial contacts three SMP proteins in ERMES (Mmm1 “1”, Mdm12 “12”, Mdm34 “34”) form a bridging complex along with the porin Mdm10 (“10”). It is not known which component binds directly to Gem1, which has been omitted for clarity. The complex is likely to transfer at least one lipid, possibly more. At ER-plasma membrane contacts three different types of SMP protein are found: E-Syts (“S”), Tmem24 (“T”) and PDZK8 (“Z”). Each SMP protein (and each E-Syt) may transfer a specific lipid. ER contacts with both the yeast vacuole (nucleus-vacuole junction) and the *trans* Golgi network can be populated by yet another SMP protein, Nvj2 (“N”), which may transfer ceramide [102]. Only ER-*trans* Golgi network contacts are shown because they are more likely to represent the site of conserved function; organisation of Nvj2 at the nucleus-vacuole junction (which is less obviously conserved) would be highly similar. Other targeting by SMP proteins includes *Arabidopsis* E-Syt2.1 (SYT5) at intracellular puncta [86], but it is not yet known whether these are contact sites or which organelles are involved. In most of these examples the lipids transferred are not yet defined clearly. In addition, if the shuttle model applies, two lipids may be exchanged in a counter-current [43].

When TULIPs are positioned exactly in the gap between two membrane-bound structures (lipoproteins or cellular organelles), they are maximally able to channel lipids between the two bodies. Inside cells, proteins with SMP domains all localise to intracellular membrane contact sites, which are common locations for LTPs in general (see Box 1) [37], [56]. This indicates that SMP proteins are well positioned to mediate net lipid exchange between the two organelles, no matter whether they function by tunneling or shuttling lipids (see above). There are at least four different contact sites targeted by SMP proteins, all involving the ER (Fig. 5). SMP domains are anchored at these precise locations by their transmembrane domains, and are presumed to directly transfer lipids in and/or out of the ER.

In human serum both CETP and PLTP are implicated in traffic of various lipids between lipoproteins. For CETP, genetics strongly support its involvement in moving cholesterol ester from HDL to LDL [67]. The “tunnel model” for CETP requires that TULIP rearranges to allow lipids to flow along its entire length, which is far from being established (see above). In addition, the tails of its TULIP hetero-dimer should simultaneously contact both donor (HDL) and acceptor (LDL). In support of this, single particle EM has visualised 1:1 complexes of CETP + HDL with 4 nm of the CETP molecule embedded [68]. Modelling the interaction between CETP and HDL by molecular dynamics can produce this degree of penetration [55], but has also suggested penetration of just 1 nm, prevented from going deeper by charged patches at or near the tips of CETP [54]. Yet more molecular dynamics using the unmodified crystal structure produce a completely different interaction, with banana-shaped CETP wrapping round HDL and lipid entering near the dimer interface (away from the tips) [47]. In addition to this model, which questions the whole tunnel model, there are two more caveats: antibodies to CETP that block lipid transfer bind to the protein's central region, not the tips, while antibodies to the tips do not block [69]; and no complex has been reported between CETP and LDL. This shows that the mode of action of CETP is yet to be established.

A further example of lipid handling outside cells is provided by the BPI-homologue NRF-5 in nematode worms. This is required for the transfer of phosphatidylserine (PS) from apoptotic cells to adjacent engulfing cells [70]. While NRF-5 can transfer cholesterol, its ability to transfer PS has not been tested, so it may act indirectly. Notably, if the action were direct, then NRF-5 would mediate transfer of lipid without being held at a contact site. This may be an extracellular example of the finding from intracellular LTPs that lipid transfer does not require direct contact, but that soluble LTPs may function less efficiently, as shown by their very high copy number per cell [71], [72].

#### 4.2.1 YceB

The YceB pre-protein has a hydrophobic sorting signal for attachment to the inner surface of the outer membrane of *E. coli* [73], followed by a short spacer (10 aa) then the TULIP domain. This positioning means that it could be involved in presenting a hydrophobic ligand to a signalling cascade in the outer membrane. Alternately, YceB might function in lipid traffic. Lipid traffic occurs across the periplasmic space of *E. coli* and all Gram-negative bacteria. At least one LTP complex forms a bridge from inner to outer membrane: the LptA/LptC/LptD complex which transfers LPS [74], so lipid traffic at membrane contacts is a prokaryotic invention (see Box 1). Other periplasmic LTPs already described include MlaC, a soluble protein with an NTF-2 fold that transfers phospholipids from outer to inner membrane [75]. If YceB also traffics lipid across the periplasmic space, its tight anchoring in the outer membrane suggests that it might act together with another (undiscovered) LTP, to hand the lipid across the whole contact site, as achieved by the Lpt and Mla complexes.

### 4.3 Providing structure to inter-organellar bridges

Since all SMP domains are targeted to membrane contact sites, they are often described as “tethers” for contact sites, a term used to imply that the proteins play a structural role in bridging. Both E-Syts and ERMES localise to contact sites that contain many other bridging proteins, so it is difficult to identify any one protein as a major structural bridge at those contacts [76]. Clearly, if these SMP proteins can bind to both sides of a contact site via accessory domains (Fig. 1B), they can be structural components. The question is how important are they to the structure of the contacts? To properly test the role of SMP proteins in providing structure at contact sites versus lipid traffic, a sensible approach is to determine if mutants that solely inhibit lipid occupancy also inhibit function, as these mutations might not affect structural composition of bridges [77].

In the case of ERMES, loss of any one member of the complex has strong effects on mitochondrial morphology, and this is rescued by expression of an artificial ER-mitochondrial structural bridge [58]. For that reason ERMES might be structurally important. However, loss of any ERMES subunit causes massive expansion of mitochondrial contacts with vacuoles (the yeast equivalent of lysosomes) [78], [79] with only minor effects on the amount of ER-mitochondrial contact [80], so ERMES is unlikely to function primarily as a structural bridge. Indeed, ERMES has a clear role in PS traffic from ER to mitochondria [81], although this is only seen if compensatory changes to loss of ERMES are mitigated by a suppressing second-site mutation [82].

E-Syts are also proposed to have a structural role at contact sites [83]. Yeast and humans each have three E-Syts at ER-plasma membrane contacts, and deletion of them all has barely any effect on contact architecture in yeast [84], but reduces contact by > 50% in humans [83]. By contrast, a direct role in lipid traffic has been hard to prove for E-Syts, with low rates of transfer achieved *in vitro* [77], [85], although there is evidence that they traffic diacylglycerol *in vivo* [77]. A third model organism where E-Syts have been studied is *Arabidopsis*. This species has 10 E-Syts (confusingly called synaptotagmins, SYTs) not 5 E-Syts as previously reported [86]. The effect on contact architecture has not been determined for deleting single plant E-Syts let alone multiple ones. However, unlike the other two species where deletions produce at most minor phenotypes, deleting just E-Syt1.1 (also called SYT1 or SYTA) has strong effects on the form and stability of the cortical ER network in leaf cells [87]. One explanation for this is that E-Syt1.1 carries out a unique structural bridging role in these cells. However, E-Syt1.1 is one of the E-Syts with the most closely related homologues: E-Syt1.2 (SYTB) and E-Syt1.3 (SYTC) are 62% and 48% identical respectively. This suggests an alternative possibility that it is the lack of lipid traffic by Esyt1.1 that causes various indirect effects, as seen for deletion of any ERMES subunit.

### 4.4 Surfactant

PLUNCs are found in various secretions that form thin films on epithelial surfaces, including the nasopharynx and the lungs. Short-PLUNC1 (BPIFA1) is one of a small group of proteins that has well characterised surfactant properties [88], [89]. Among the many varied PLUNC proteins, horses have a unique short-PLUNC1 homologue called latherin, which owes its name to being the major component of the white sweat (or lather) that appears on the coats of horses when they sweat profusely. The surfactant action of latherin produces a large air-water interface for heat loss during galloping [90]. To act as a surfactant, latherin and other short-PLUNCs must cover a large area at a water (hydrophilic)-air (hydrophobic) interface. Structural studies on latherin [40] and short-PLUNC1 [91] have produced two models for how the proteins could change their conformation from an almost solid cylinder to something with a greater area. One idea is that the whole super-roll flattens out (Fig. 2C–> 2B–> 2A), but this seems energetically unlikely [40]. A different idea focusses on the loop between the 1st and 2nd elements, which is unique to this group of short-PLUNCs. The loop in short-PLUNC1 is highly unstructured (9 out of 25 residues are glycines) and contains four leucines that are required for surfactant activity [92]. This suggests that the loop can open out massively to increase the protein's surface area by almost double, with all four leucines contributing to the water-air interface. This may not be the only conformation, since the loop is weakly predicted to form a helix, which could fold up against the rest of the domain. Surfactant activity does not exclude lipid handling since short-PLUNC2 solubilises dipalmitoyl-PC, a lipid uniquely enriched in pulmonary surfactant [91].

### 4.5 Protein–protein interactions that affect epithelial functions: a link to cystic fibrosis

All TULIPs may engage in protein–protein interactions as well as protein–lipid interactions., but only a few have been studied so far. Short-PLUNC1 not only acts as an abundant surfactant and binder of dipalmitoyl-PC, but it also has a third action on epithelia that is mediated by a protein-protein interaction: it directly binds the epithelial sodium channel (ENaC). This inhibits ENaC activity, which is to internalise sodium and with it water, so short-PLUNC1 causes increased wetting of the epithelial surface [93]. The inhibitory binding of short-PLUNC1 to ENaC is sensitive to acidic environment and drops sharply between pH 7 and pH 6. Importantly, pulmonary secretions are maintained at neutral pH by the chloride/bicarbonate exchange activity of the cystic fibrosis transmembrane conductance regulator (CFTR). Therefore, in cystic fibrosis patients, the acidification that arises from CFTR inactivity leads to decreased short-PLUNC1 binding to ENaC, and hence the airway surface dries out [94]. This model fits with PLUNC transcripts being up-regulated in cystic fibrosis [95].

Another protein-protein interaction of short-PLUNC1 is with the store-operated Ca^2+^ entry channel Orai1, which it binds using different residues from those that bind ENaC. The interaction inhibits Orai1, so that in its absence there is increased entry, increased smooth muscle contractility, and bronchoconstriction [96]. Interestingly, sputum levels of short-PLUNC1 are selectively reduced in asthmatics compared to people with or without other respiratory problems. These studies show that other actions of TULIPs can arise from protein-protein interactions, possibly completely independent of lipid handling.

### 4.6 Fibroin p25 – a fatty twist in the story of silk

**Fig. 6:**
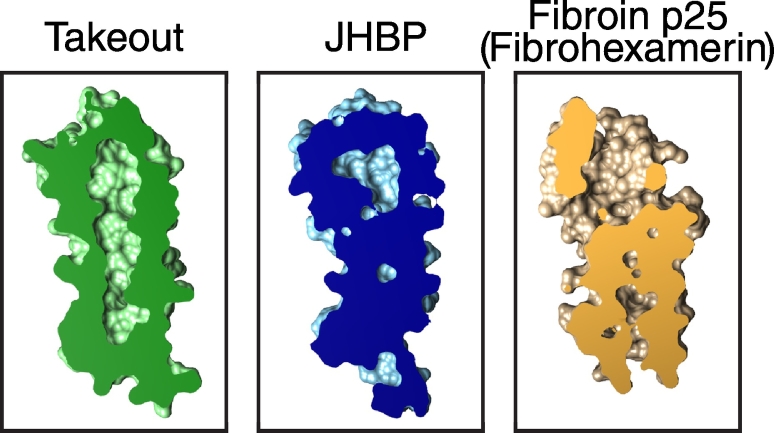
Prediction of a lipid binding cavity in fibroin p25. A model of fibroin p25 was based on 3e8t (Takeout) and 3aos (JHBP) and made with Modeller [103] as applied by HHpred [26]. The model is available as Supplementary File 1. Cross-sections through the two known structures and the model (visualised in Chimera) show that cavities in p25 are intermediate in size between Takeout and JHBP, indicating that its side-chains are not significantly larger than either documented LTP. Hence, p25 is likely to have a cavity large enough for it to act as an LTP. Prediction of a lipid binding cavity in fibroin p25. A model of fibroin p25 was based on 3e8t (Takeout) and 3aos (JHBP) and made with Modeller [103] as applied by HHpred [26]. The model is available as Supplementary File 1. Cross-sections through the two known structures and the model (visualised in Chimera) show that cavities in p25 are intermediate in size between Takeout and JHBP, indicating that its side-chains are not significantly larger than either documented LTP. Hence, p25 is likely to have a cavity large enough for it to act as an LTP.

Silk-producing caterpillars of the mulberry silkworm (*Bombyx mori*) and related moths secrete silk from specialised glands that are embryonically related to salivary glands [97]. This silk is made from fibroin fibres embedded in the serine-rich short protein sericin. Fibroin itself consists of three proteins in 6:6:1 ratio: 6 × fibroin heavy chain (> 5000 aa), which makes up the long highly repetitive fibrous material, 6 × fibroin light chain which binds to the C-terminus of the heavy chain, and 1 × p25, a 220 aa protein named for its molecular mass of 25 kD. p25 is needed for formation of the complex [98], hence its alternative name of fibrohexamerin. p25 is annotated in 29 proteins in NCBI, all in moths and butterflies, and previously nothing has been reported on its structure. We found that it has full-length homology with JHBP that is detectable by PSI-BLAST from the 2nd iteration onwards (Supplementary Fig. 1). A structural model of p25 indicates that side-chains of residues lining the lipid binding cavity are small enough to fit a lipid inside (Fig. 6). The significance of a component of silk being a TULIP is unclear. One possibility is that p25 provides a negative feedback mechanism via juvenile hormone, which stimulates heavy chain production [99]. If p25 binds JH, then increased production of silk down-regulates free JH and so turns down silk production. Another possibility is that p25 binds the natural pigments that lend cocoons distinct colours, mixtures of carotenoids and flavonoids. These ideas might be tested directly by lipid solubilisation assays with p25.

### 4.7 Multiple LTP domains in single proteins

What is the significance of the widespread occurrence of dimerised TULIP domains, particularly their encoding in the same protein? This question has not been directly studied, but its relevance goes beyond TULIPs, as other LTPs occur in multiple copies per protein. Some LAM proteins have pairs of StAR-kin domains [100]. There are fungal proteins with three StAR-kin domains, for example the *Ustilago maydis* protein UM05383. Other proteins combine LTP domains from multiple superfamilies: CRAL/TRIO plus PRELI in Sec14-like 1/5, and LAM plus DUF3074 in some fungal proteins. Assuming that the two LTP domains are functionally linked, there are several hypotheses to choose between. If the two domains transfer the same lipid, which is most applicable for SMP homodimers, then if the domains work in parallel the role for dimerisation is not obvious. This leaves the alternative that multiple domains work in series, with lipid being handed on from one to the next, which is consistent with either a tunnel or a series of shuttles. Although there is no evidence to support this mode of action for TULIPs, it does apply to LPS transfer by the LptA/C/D complex in bacteria, where four domains create a continuous structure (“slide”) for LPS to cross the periplasmic space [74].

However, this explanation cannot apply to all cases of multiple LTP domains. For Lam2 in yeast, we found that an activity associated with lipid traffic required just one of the two StAR-kin domains, even though the other domain bound the same lipids and was active when expressed alone [100]. Similarly, only the N-terminal TULIP in BPI binds LPS tightly, while the C-terminal domain interacts with CD14 [101]. Therefore, different domains may have different actions, which could include: moving the same lipid in different directions or at different contact sites; different (even if overlapping) lipid specificity; division of function between lipid and protein binding (as in BPI); and even regulation of one domain by another.

## 5 TULIP evolution

The group of full length homologues of YceB, with the same domain structure but diverged sequences, has been named the domain of unknown function (DUF)-1439. DUF1439s are predominantly found in gamma– and beta–proteobacteria, but they are also present in other phyla, including fusobacteria, actinobacteria, bacteroidetes, aquificae and even a cyanobacterium. This finding of TULIPs in a diverse group of prokaryotes indicates that previous considerations that TULIPs evolved only in eukaryotes have missed pieces of the puzzle [20]. One explanation for the wide dispersal of TULIPs in bacteria is that there was one single progenitor DUF1439 in the last universal common ancestor with multiple losses, though repeated horizontal transfer cannot be excluded.

The widespread presence throughout eukaryotic evolution both of TULIP-N/TULIP-C pseudo-dimers and of SMP proteins of the four major types (Fig. 1B) implies that all these were present in the last eukaryotic common ancestor, and that the ancient duplication in the BPI lineage occurred before this stage, not afterwards as previously suggested [20]. Dimerisation within the CETP/BPI domain and between SMP domains indicates that this did not evolve twice, but that their common ancestor, the “proto-TULIP”, had this capacity, which has been selectively lost in some proteins (for example short-PLUNC and JHBP) [20]. Since TULIP dimerisation pre-dates the emergence of the last eukaryotic common ancestor, we suggest that dimerisation may have occurred in prokaryotes, although there is no evidence for that yet. More information may come to light when bacterial and archeal sequences are mined more deeply for evidence of relatives of YceB.

## 6 Conclusion

Before 2010, TULIPs were a well-known family of extra-cellular proteins involved in lipid handling in eukaryotes, but since then they have been found inside these cells and other cells. There are several questions for the near future. One is whether the “tunnel model” can be supported by firm observations, some of which will be structural studies of the extreme tail of TULIPs to look for rearrangements that allow the formation of pores missing from current crystal structures [29], [68]. So far such rearrangement is confined to a molecular dynamics simulation [54]. This should not take away from answering more general questions about the role of protein flexibility in binding and transferring lipids, which might allow lipids to bind inside proteins where crystals do not reveal large cavities (for example dipalmitoyl-PC in short-PLUNC1). Another set of questions surround models of dimerisation and multimerisation, especially of ERMES subunits, to determine if and how a multitude of LTP domains collaborate together in the traffic of one lipid. Finally, since the existence of many TULIPs was not suspected until the structures became apparent through structural studies, we predict that many more TULIP varieties exist. One key way to discover new TULIPs will be to solve more structures. But it is also important to work out where to look among the many proteins domains. This will rely on increasing the sensitivity of profile-profile tools even more.

The following are the supplementary data related to this article.

Supplemental Fig. 1 PSI-BLAST evidence that Fibroin is a homologue of JHBP.
Fibroin p25 sequence from *Bombyx mori* was submitted to PSI-BLAST at NCBI for two iterations, producing 59 hits with E-values ≤ 10 that are annotated either as fibroin p25 or as JHBP/Takeout, and 34 other hits with no or other annotations. A: e-values of the most significant 59 annotated hits. All 29 fibroin p25 hits are much lower more significant than the 30 JHBP/Takeout hits. Inset: expansion of indicated region, showing that the most significant JHBP/Takeout hit has an e-value *p* < 0.001 (− log_10_ of e-value > 3). B: details of least significant hit to an annotated fibroin p25 (hit #29). C: details of most significant hit to an annotated JHBP/Takeout (hit #30). Hit 29 is strong across 70 residues, while hit 30 is weaker but persists across 150 residues. In further PSI-BLAST interactions, the hit list becomes dominated by JHBP/Takeout, which outnumber p25 65-fold. Image 1

Supplemental Movie 1 YceB is a structural homologue of the C-terminal TULIP domain of BPI.
Rotation of YceB (from 3l6i, coloured as in Fig. 3D) aligned with the C-terminal domain of BPI (from 1 bp1, coloured white) and its associated lipid (from 1 bp1, standard colours, partly transparent). For clarification, segments of the movie also show both BPI alone (after 23 s) and YceB alone (after 34 s). Alignment made and visualised by CCP4MG (version 2.10.4). Supplemental Movie 1

Supplemental File 1 PDB file of model of *B. mori* fibroin p25
PDB file of a model of fibroin p25 from *Papilio xuthus*, the Chinese yellow swallowtail butterfly. HHpred was initiated with the full sequence (Genbank BAB39504.1, 215 aa), with 8 iterations of PSI-BLAST used to make a multiple sequence alignment. The two strongest matches among solved structures were 3e8t_A (Takeout) and 2rck_A (JHBP), with e-values of 10^− 34^ and 10^− 29^ respectively. To elongate matches, matching was repeated with “MAC realignment threshold” set to 0.2, leading to matches at 184 columns in both cases. Modeller was then applied using these two structures as templates. In the model the first 25 residues, form an extended loop, consistent with the prediction that they form a signal sequence. These were excluded from subsequent work defining the cavity (Fig. 6). Supplemental File 1

## Transparency document

Transparency document. Image 2
