# Supplementary material for: Tubular lipid binding proteins (TULIPs) growing everywhere
Source: Biochim Biophys Acta. 2017 Sep;1864(9):1439–49. doi: 10.1016/j.bbamcr.2017.05.019 (PMC5507252; doi:10.1016/j.bbamcr.2017.05.019)
Supplement: Supplemental Fig. 1 — PSI-BLAST evidence that Fibroin is a homologue of JHBP. Fibroin p25 sequence from Bombyx mori was submitted to PSI-BLAST at NCBI for two iterations, producing 59 hits with E-values ≤ 10 that are annotated either as fibroin p25 or as JHBP/Takeout, and 34 other hits with no or other annotations. A: e-values of the most significant 59 annotated hits. All 29 fibroin p25 hits are much lower more significant than the 30 JHBP/Takeout hits. Inset: expansion of indicated region, showing that the most significant JHBP/Takeout hit has an e-value p < 0.001 (− log10 of e-value > 3). B: details of least significant hit to an annotated fibroin p25 (hit #29). C: details of most significant hit to an annotated JHBP/Takeout (hit #30). Hit 29 is strong across 70 residues, while hit 30 is weaker but persists across 150 residues. In further PSI-BLAST interactions, the hit list becomes dominated by JHBP/Takeout, which outnumber p25 65-fold. [file mmc1.pdf]

Supplementary Figure 1

A

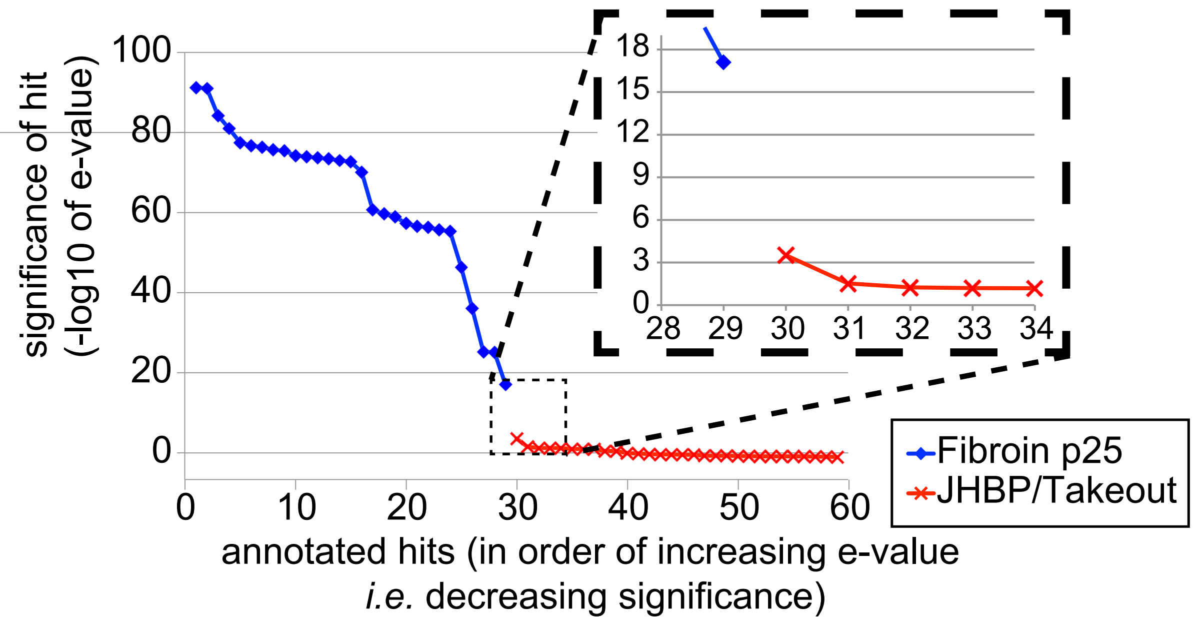

B

Hit 29: KOB69468.1 Fibroin P25, partial [Operophtera brumata]
Length=184

Score = 87.1 bits (214), Expect = 8e-18, Method: Composition-based stats.
Identities = 21/72 (29%), Positives = 32/72 (44%), Gaps = 0/72 (0%)

```
Query  18    GPPSPIYRPCYLDDYKCISDHLAANSKCIPGRGQIPSQYEIPVFQFEIPYFNATYVDHNL  77
             G    + RPC ++DY+CI    A NS+C P  G  P    +      +PY N TY   N+
Sbjct  112   GTREDLVRPCQMEDYQCIRKLFAENSQCNPSYGPSPDPLYLDQNTMHLPYINLTYTLTNV  171

Query  78    ITRNHDQCRVSE  89
                     ++ +
Sbjct  172   KVSGMASAKIID  183
```

C

Hit 30: XP_011562916.1 PREDICTED: juvenile hormone-binding protein-like isoform X2 [Plutella xylostella]
Length=245

Score = 51.2 bits (121), Expect = 3e-04, Method: Composition-based stats.
Identities = 31/175 (18%), Positives = 58/175 (33%), Gaps = 15/175 (9%)

```
Query  7     AVAAVAVLASAGPPS-PIYRPCYLDDYKCISD----HLAANSKCIPGRGQIP-SQYEIPV  60
              +  +     A P +  I   C  +D +C+ +     L   S+ IP     P     IP
Sbjct  8     CLFVLVGCVFAKPEAGSIASVCNAEDEECVKESLKTFLRTTSQGIPAFDVRPLDPNVIPR  67

Query  61    FQFEIPYFNATYVDHNLITRNHDQCRVSEFYDNVRTLKTVLTVDCPWLNFESNRTLAQHM  120
               + +  F   Y  +NL        R++E+  +     +++  +   L++ S+  +
Sbjct  68    INYTLGAFGVGYTFNNLTVTGMKNLRLAEYNIDRAAKTSLIKTEAT-LDYVSDVEIDYLK  126

Query  121   SFKEDVVLSFYINGSYPLIR------LTTVFDKGNNFDLCSAFTFADLAGGLPIF  169
               K  V    Y      L+       +TT      +FD+       D       F
Sbjct  127   LGK--VFTGKYTGAGKALVTVKYPYGITTDDKGVQHFDVQQETIECDPVEASGGF  179
```
